# Primary Closure of Pilonidal Sinus With Slide-Swing Skin Flap Compared With the Secondary Closure: A Single-Blinded Randomized Controlled Trial

**DOI:** 10.7759/cureus.32880

**Published:** 2022-12-23

**Authors:** Hormoz Mahmoudvand, Sedighe Nadri, Ramin Shekouhi, Maryam Sohooli, Seyed-Ahmad Seyed-Alagheband

**Affiliations:** 1 Department of Surgery, Lorestan University of Medical Science, Lorestan, IRN; 2 Department of Anesthesiology, Lorestan University of Medical Science, Lorestan, IRN; 3 Colorectal Research Center, Shiraz University of Medical Sciences, Shiraz, IRN

**Keywords:** slide-swing skin flap, limberg flap, secondary closure, flap technique, pilonidal sinus disease

## Abstract

Introduction: There are many surgical approaches for sacrococcygeal pilonidal sinus disease (PSD) therapy, ranging from wide excision repair to less morbid excisions with primary/flap closure. The off-midline flaps, which shift the incision line away from the midline natal cleft, have been associated with lower recurrence rates than the conventional mid-line closure techniques. This single-blinded randomized controlled trial aims to compare the short/long-term efficacy and outcome of the slide-swing flap technique with the conventional secondary wound closure.

Method and materials: This study was a prospective randomized controlled trial conducted on patients with PSD. Patients were assigned into two groups: secondary closure (control) and slide-swing flap (trial). Patients were advised to visit the surgical clinic two times weekly for the two weeks after the operation for at least six months.

Results: In this study, 100 patients were enrolled. They were assigned into two groups of control, and trial. The mean age of all participants was 29.15 ± 8.36 years old (age range: 18-62 years old). The mean operation time was 39.65 ± 12.63 for both groups, with the control group being 29.70 ± 7.71 and the swing flap group 46.90 ± 7.81. Patient visual analog scale (VAS) scores in both groups revealed that the trial group was associated with lower VAS scores compared with patients who underwent secondary closure (p-value = 0.006). Also, the trial group demonstrated a higher rate of healing, better cosmetic outcomes, and quicker recovery time compared with the controls.

Conclusion: Compared with secondary closure, the slide-swing flap was associated with excellent cosmetic outcomes, disease recurrence, and recovery time. Also, the post-operative complications were significantly lower compared with the traditional method.

## Introduction

Sacrococcygeal pilonidal sinus disease (PSD) is considered a chronic inflammatory disease with a significant male predominance [1]. Traditionally, the main cause of PSD occurrence was thought to be congenitally driven. But now, accumulation of in-growing hair in natal cleft and subsequent inflammatory reactions is the main pathogenesis behind PSD [2]. Various predisposing factors such as obesity, chronic irritation (especially prolonged sitting position), and poor hygiene are associated with PSD occurrence, which reduces lifestyle quality of patients tremendously [3]. Early-onset treatment is a necessity for patients with PSD. Needless to say, without proper treatment PSD can persist for years and progress to multiple painful puss-secreting skin openings that will eventually requires wider excisional surgery with higher chance of complications [4].

Surgical management of PSD is considered the mainstay treatment option with variable rate of recurrence. There are many surgical approaches for PSD therapy, ranging from wide excision repair to the less morbid excisions with primary/flap closure [5]. The optimal surgical treatment option should rely on complete sinus extraction with low recurrence rate, rapid recovery time, and minimum cosmetic-associated morbidities [6-7]. The off-midline flaps, which shifts the incision line away from the midline natal cleft, have been associated with lower recurrence rate compared with the conventional mid-line closure techniques [8-9]. Furthermore, the asymmetric-oblique closure of PSD or flap techniques have proven to be less associated with disease recurrence [8]. Various flap techniques are available for surgical management of PSD.

For instance, the Limberg flap is a popular off-midline surgical technique with the principle of flap mobilization and recurrence rate of less than one percent [10]. The flap mobilization associated with this technique results in less dead space following sinus excision and prevents subsequent midline tension, which ultimately leads to lower recurrence rate [11]. Similarly, the Karydakis procedure is another off-midline surgical flap that showed promising results in the literature. Although both of these methods are effective procedures for management of PSD, they are also associated with some limitations. For instance, the inferior pole of the Limberg flap stays in the intergluteal sulcus, which may lead to subsequent prolonged wound healing and infection. Also, the Karydakis procedure is limited in operating PSD with sinuses that are far from the midline. Thus, we conducted this single-blinded randomized controlled trial to compare the short/long term efficacy and outcome of slide-swing flap technique with the conventional secondary wound closure.

## Materials and methods

Study protocol

**Figure 1:**
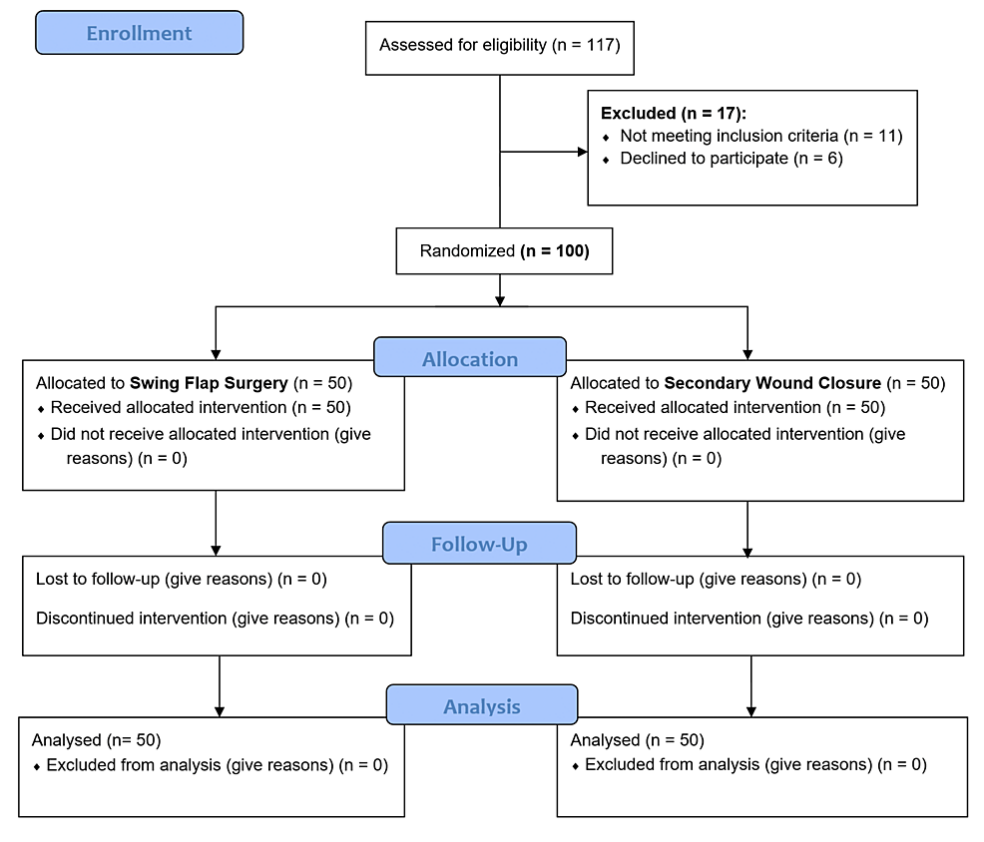
The CONSORT flowchart of this study.

This study was a prospective randomized controlled trial conducted on patients with PSD. The study was carried out in the department of surgery, Lorestan university of medical sciences from March 2021 until February 2022. This study was approved by the institute ethical committee with approval number of IR.LUMS.REC.1399.259. Also, our study was registered at Iranian Registry of Clinical Trials with registration number of IRCT20210926052604N1. Inclusion criteria for this study were all adult (age ≥ 18 years old) patients with diagnosis of PSD (via physical examination) that could tolerate surgical operation. Accordingly, patients with American Society of Anesthesiology (ASA) score of three and four were excluded. Also, patients with age of less than 18, pregnancy, immunodeficiency, suppurative hidradenitis of sacral region, and patients who declined to participate in the study were excluded. A total of 117 patients were evaluated initially for the study, in which 17 patients were excluded from the study (Figure 1). Finally, by considering a two-sided type-I error at 0.05, a total sample size of 100 patients was calculated [12]. Prior to patient’s randomization, all patients were advised about the surgical operations by the surgical team. A written informed consent was obtained from all participants. It is noteworthy to mention that all surgical procedures were conducted by an experienced surgeon in order to eliminate the risk of observer's bias.

Randomization and blinding

The randomization process was carried out by a computer software. Patients were assigned to either the slide-swing skin flap (trial group) or the secondary closure (control group). Data gathering after the procedure was conducted by research fellows of the institute, which they were also blinded from the study groups in order to decrease the risk of bias.

Surgical procedures: Prior to surgery, all patients were assessed by both teams of anesthesiology and surgery. All participants underwent thorough laboratory workup and preoperative assessment. Patients were operated via spinal anesthesia. All participants received IV antibiotic (1-g cephazolin) prior to the surgical operation. The surgery was conducted by a single surgeon in jackknife position with buttocks being retracted.

Control Group (Secondary Closure)

After pre-operative assessments, the involved area was carefully incised down to the sacral fascia. All pilonidal sinuses were excised and checked afterwards for any remnants. After irrigation of the surgical site, the wound was left open for secondary closure. 

Trial Group (Swing Flap)

**Figure 2:**
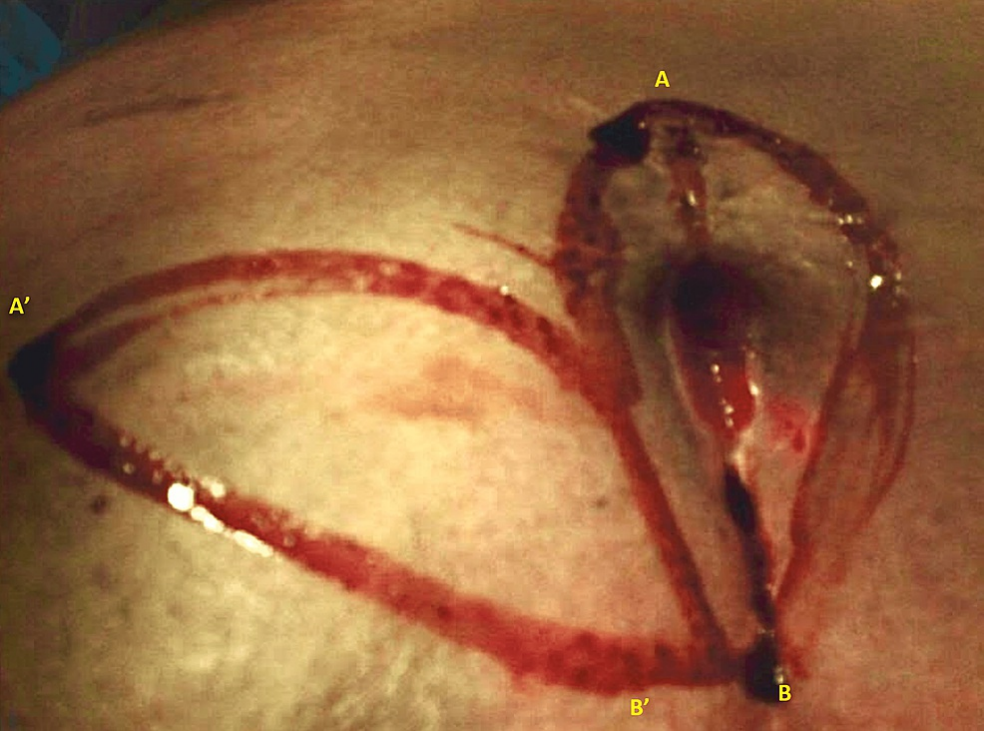
Incision borders in the slide-swing skin flap.

**Figure 3:**
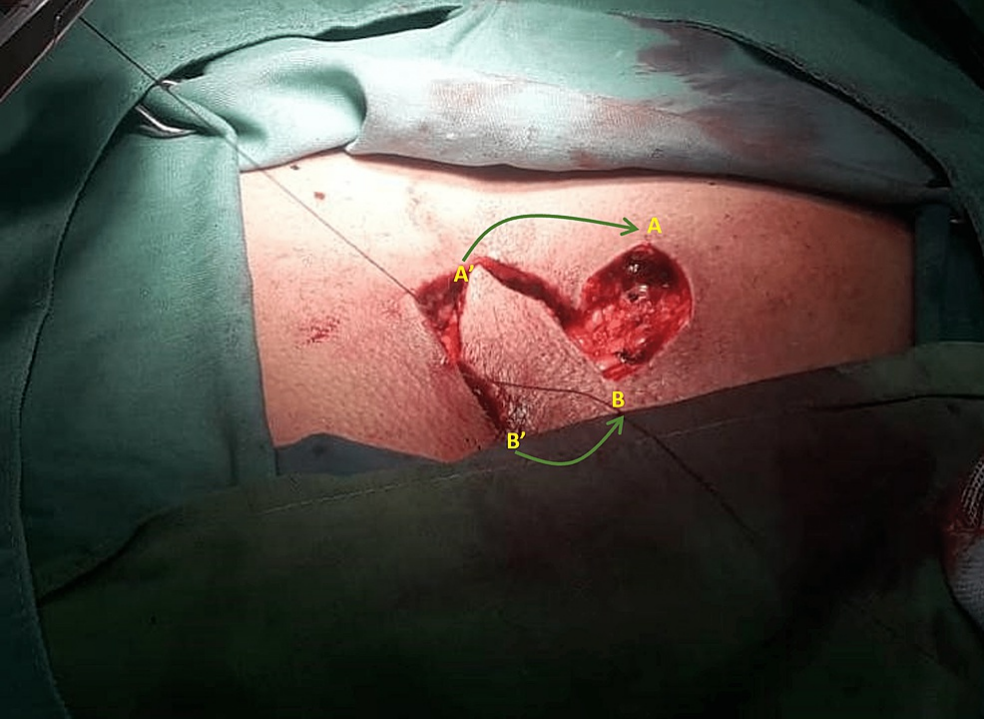
After sinus removal, the surgical wound edges were mobilized and swing flap was made with subsequent closure.

**Figure 4:**
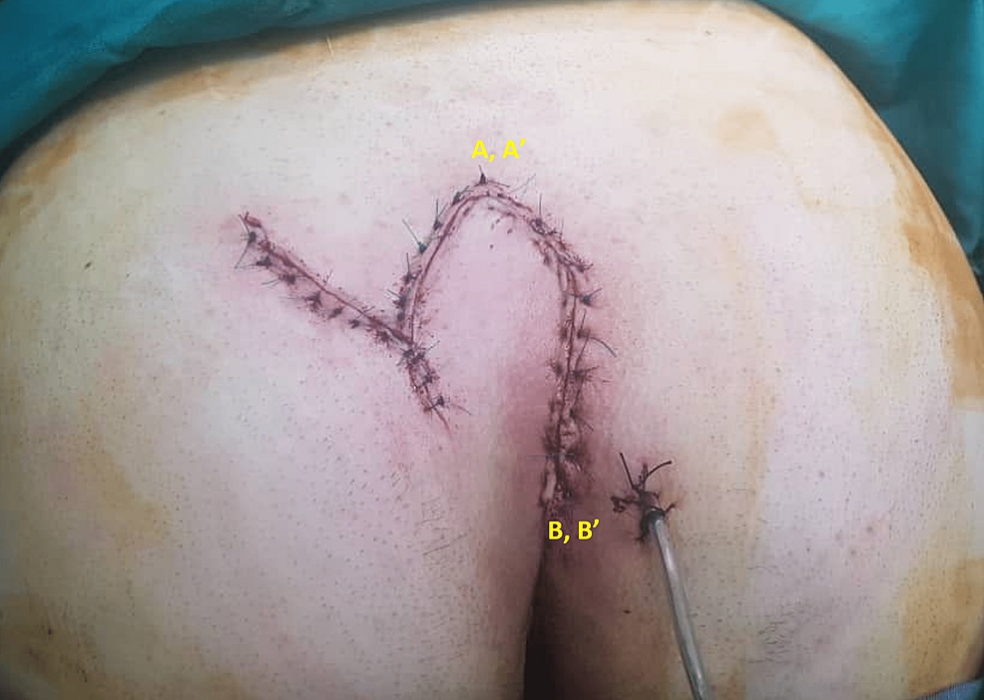
Surgery site after the slide-swing skin flap technique.

After pre-operative evaluations, two off-midline elliptical incisions were made at the involved area encompassing all pilonidal pits. As shown in Figure 2, measurements of both length and width of the incision were made. Another incision was made with the same length and width for the flap. The involved area was incised down to the sacral facia and the hemostasis was provided with the aid of electrocautery. All sinuses were excised down to the erector spinae muscle aponeurosis layer and checked for complete removal of sinuses. After sinus removal, the surgical wound edges were mobilized and swing flap was made with subsequent closure (Figure 3). A suction drain was placed afterwards (Figure 4). The suction was removed the next day when discharge was below 20 mL/d. Standard post-operative care was carried out for both groups of control and trial. Patients were ambulated and allowed for orals 4 h after the surgery. Patients were discharged from the hospital with oral antibiotic and hygienic recommendations.

Variables

Data gathering was performed regarding patient’s age, sex, body mass index (BMI), duration of PSD before surgery, previous medical disease, operation type, operation time (operation period was measured from incision time to the end of wound closure), and duration of hospitalization (day of surgery was assumed as day zero).

Follow-up sessions

Patients were advised to visit the surgical clinic two times weekly for the two weeks after the operation for at least six months. During each follow-up visit, patients were evaluated by the same surgeon for degree of pain at surgical site, hematoma and seroma formation, signs of infection, wound dehiscence, scar formation, duration of incapacity for work, and disease recurrence. For evaluation of pain, we used the visual analog scale (VAS score). The VAS score was interpreted as mild pain with score of 0-3, moderate pain with score of 4-6, and severe pain with score of 7-10. Also, for cosmetic outcomes we used the VAS score and the interpretation is shown in Table 1. Scar formation was defined as complete re-epithelialization of surgical site and was the factor attributed to cosmetic outcome.

**Table 1:** Comparisons between the two groups in terms of healing, recurrence, and complications. VAS, visual analog scale *VAS score interpretation: 0-3 = minimum satisfaction; 4-6 = moderate satisfaction; 7-10 =complete satisfaction ^1^Chi-Square test; ^2^Independent t-test

Variable	Total (100, 100.0%)	Control (50, 50.0%)	Trial (50, 50.0%)	p-value^1^
Infection				
At 1 week	17, 17.0%	9, 18%	8, 16%	0.79^1^
At 2 weeks	3, 3.0%	1, 2.0%	2, 4.0%	0.55^1^
At 4 weeks	0, 00.0%	0, 00.0%	0, 00.0%	-----
Hematoma				
At 1 week	22, 22.0%	5, 10.0%	17, 34.0%	0.003^1^
At 2 weeks	3, 3.0%	0, 00.0%	3, 6.0%	0.07^1^
Seroma				
At 1 week	74, 74.0%	49, 98.0%	25, 50.0%	<0.001^1^
At 2 weeks	64, 64.0%	46, 92.0%	18, 36.0%	<0.001^1^
At 4 weeks	25, 25.0%	23, 46.0%	2, 4.0%	<0.001^1^
Complete healing				
At 3 months	73, 73.0%	32, 64.0%	41, 82.0%	0.04^1^
Cosmetic satisfaction				
VAS score* (mean)	100, 100.0%	8.08 ± 1.49	9.04 ± 1.52	0.002^2^
Wound dehiscence	3, 3.0%	0, 00.0%	3, 6.0%	0.79^1^
Duration of incapacity for work (days)	29.89 ± 13.31	34.90 ± 15.72	24.88 ± 10.91	<0.001^2^
Recovery (healing) time (days)	54.36 ± 20.85	81.6 ± 33.5	27.12 ± 8.21	<0.001^2^
Recurrence				
At 3 months	3, 3.0%	2, 4.0%	1, 2.0%	0.55^1^
At 6 months	2, 2.0%	2, 2.0%	0, 00.0%	0.15^1^

Statistics

The authors used SPSS software version 25 (IBM Corp., Armonk, NY) for descriptive analysis of patient’s baseline demographics and other variables. The data were analyzed based on frequency and frequency percentage. Quantitative variables were reported as mean ± standard deviation (SD) and qualitative variables were reported as numerical (percentage) data. The Kolmogorov-Smirnov test was conducted for evaluation of data normality, and subsequently the independent sample t-test was performed as a parametric test, and Mann-Whitney U test as a non-parametric test. Also, Chi-square test for evaluation of qualitative variables. A linear regression analysis was performed to evaluate any possible association between the patient's outcomes and clinical characteristics. A p value of lower than 0.05 was assigned as significant.

## Results

Baseline demographic information of patients

In this single-blinded randomized controlled trial (RCT), 100 patients were enrolled. They were assigned into two groups of control (50, 50.0%) and trial (50, 50.0%). The mean age of all participants was 29.15 ± 8.36 years old (age range: 18-62 years old). Table 2 shows baseline demographic characteristics of patients in both control and trial group. Accordingly, there are no significant difference between the two groups of trial and control in terms of age, sex, BMI, and past medical history (Table 2). However, patients in the flap (trial) group showed lower duration of PSD prior to surgery compared with the secondary flap group (p-value=0.04). In order to evaluate the risk of confounder bias, we performed a stepwise multiple regression analysis investigating the possible effects of PSD duration on the outcome measures. Accordingly, there were no significant associations between PSD duration and patient's outcome, recurrence, hematoma, and seroma formation. Figure 1 shows a flow chart of patient’s allocation in both groups. The mean operation time was 39.65 ± 12.63 for both groups, with the control group being 29.70 ± 7.71 and the swing flap group 46.90 ± 7.81. The difference between the operation time that was observed in the control group when compared with the trial group was statistically significant (p-value=0.000). As demonstrated in Table 2, the mean time of hospitalization was less among the trial group compared with the control group. However, the observed finding was not statistically significant (p-value=0.066).

**Table 2:** Baseline demographic characteristics of patients in both groups. BMI, body mass index; PSD, pilonidal sinus disease; M, male; F, female; SD, standard deviation ^1^Chi-Square test; ^2^Independent sample t-test

Variable	Total	Control
Sample size (n, %)	100 (100.0%)	50, 50.0%
Age (mean ± SD)	29.15 ± 8.36	28.9 ± 6.99
Sex (M; F)	85; 15	40; 10
BMI (mean ± SD)	25.54 ± 3.29	25.49 ± 3.19
Past medical history (n, %)	6 (6%)	2 (33.3%)
PSD duration (days)	325.10 ± 390.39	404.98 ± 451.83
Operation time (minutes)	39.65 ± 12.63	29.70 ± 7.71
Hospitalization time (hours)	24.48 ± 6.53	23.28 ± 4.47

Patients' VAS score after slide-swing flap compared with the secondary closure

Patients' VAS score in both groups is shown in Table 3. Our results revealed that the overall average VAS score decreased from 5.46 ± 2.33 in the first day of post-operation to 3.93 ± 1.43 at the end of two weeks. Also, thorough out the entire follow-up period, the trial group was associated with lower VAS score compared with patients who underwent secondary closure. According to Table 3, the difference in VAS score between the two groups in the first week was statistically significant (p-value = 0.006). Additionally, we observed that 46 (92.0%) of the trial group complaints of none-to-mild pain, was significantly higher than the control group (p-value = 0.029).

**Table 3:** Patients' VAS score following surgery in both groups of control (secondary closure) and trial (swing flap). VAS, visual analog scale *1 D, after one day, 1 W, after one week, 2 W, after two weeks ^1^Chi-Square test VAS score interpretation: 0-3 = mild, 4-6 = moderate, 7-10 = severe

Variable		Control (50, 50.0%)	Trial (50, 50.0%)	p-value^1^
Pain				
VAS score (1 D) *				
	Mild (n, %)	20, 40.0%	29, 58.0%	0.07
	Moderate (n, %)	21, 42.0%	17, 34.0%	0.41
	Severe (n, %)	9, 18.0%	4, 8.0%	0.13
VAS score (1 W) *				
	Mild (n, %)	25, 50.0%	40, 80.0%	0.002
	Moderate (n, %)	20, 40.0%	7, 14.0%	0.003
	Severe (n, %)	5, 10.0%	3, 6.0%	0.46
VAS score (2 W) *				
	Mild (n, %)	38, 76.0%	46, 92.0%	0.02
	Moderate (n, %)	10, 20.0%	4, 8.0%	0.08
	Severe (n, %)	2, 4.0%	0, 00.0%	0.15

Complications after slide-swing flap compared with secondary closure

According to Table 1, 17 (17.0%) patients were diagnosed with surgery site infection in the follow-up sessions. There were no statistically significant differences among both groups in terms of infection at first week follow-up (p-value = 0.791). At the second week follow-up, surgery site infection of 14 patients had already resolved. Accordingly, no new cases of infection at site of surgery were observed after the first week and all patients were infection-free at the end of one month follow-up. In terms of hematoma formation, the swing flap group was associated with statistically significant higher number of hematomas at the first week (p-value = 0.003). Additionally, almost all hematomas resolved at the end of the second week (Table 1). Furthermore, seroma formation was observed in 74 (74.0%) patients in both groups at the first follow-up session. There was a statistically significant difference between both groups in terms of seroma formation in all follow-ups. Accordingly, patients in the swing flap group were less susceptible to seroma formation compared with the traditional secondary method (Table 1).

Cosmetic outcome, recovery, and disease recurrence

Patients were assessed at three-month follow-up session for evaluation of healing. Accordingly, the trial group demonstrated higher rate of healing compared with the controls. This finding was statistically significant (Table 1). The mean cosmetic VAS score in the slide-swing flap was 9.04 ± 1.52, which compared with the secondary closure (mean VAS score of 8.08 ± 1.49), showed significant difference (p-value = 0.002). Also, our study revealed 3% frequency rate of wound dehiscence, which all patients were in the trial group (p-value = 0.794). Furthermore, recovery was significantly quicker in the trial group compared with the control group. This finding was also statistically significant. Accordingly, we evaluated patients for recurrence at three- and six-months follow-ups. At the three-month follow-up session, three (3.0%) patients had recurrence of the disease after surgery, among which only one patient was in the flap group. Also, at the six-month follow-up there were no new cases of recurrence. It is noteworthy to mention that the one patient at the trial group which was previously diagnosed with recurrence at the three-month session, was disease-free at the six-month follow-up without any surgical treatment. The observed finding was not statistically significant (Table 1). 

Stepwise linear regression of recovery time related to clinical measures (age, gender, BMI, PSD duration, and operation time) indicated that in the control group, age was positively related to recovery time (B=1.509, t=2.298, df= 48, p=0.026). No clinical variables were associated with recovery time in the trial group. In addition, logistic regression of infection related to the mentioned clinical measures indicated that BMI was related to infection in the trial group (EXP(B)=1.401, wald statistics= 6.997, df=1, p=0.008); meaning that the higher BMI increases, the likelihood of having infection in the trial group increases as well. There was also a significant relation between operation time and infection in the control group (EXP(B)=1.230, wald statistics=7.223, df=1, p=0.007). However, there were no associations between hematoma and recurrence with the patient’s clinical characteristics in both control and trial groups.

## Discussion

Despite recent advancements in PSD managements, the gold standard surgical technique remains unclear. The ideal surgical method should be characterized with eradication of the primary disease, as well as eliminating the risk of recurrence with minimal cosmetic adverse effects. In this RCT we described a modified swing-flap surgical technique for treatment of PSD. We demonstrated that compared with secondary closure, this technique is associated with reduced post-operative pain and complications, as well as faster recovery time and desired cosmetic results.

Slide-swing skin flap technique is considered a transposition flap first described in 1981 by Schrudde and Petrovici [13]. Slide-swing plasty was originally used for closure of large defects, especially after excision of benign/malignant skin tumors. Unlike Limberg flap, it has the ability to close down large round and even oval to hemispherical skin defects. Limberg flap on the other hand, requires wide excision of healthy skin that mostly change the geometric figure of the skin [13]. Another beneficial point of swing technique is the fact that the incision line could be adjusted to the defect. Thus, the subsequent scar would be much smaller compared to other flap methods. Also, the technical simplicity of swing flap along with its short operation time compared with the Limberg flap, makes this surgical method to be the superior technique.

Our study revealed that the operation time in swing flap was significantly longer compared with secondary closure (p < 0.001). In line with our results, previous studies made similar conclusions. Maghsudi et al. [14], described the operation time of secondary closure to be 13.8 ± 2.4 min, which compared with the Limberg flap (mean duration: 40 ± 3.2 min) was significantly shorter. Unfortunately, this short-duration feature of secondary closure has few drawbacks. Requirement for changing the wound dressing frequently, longer hospital stay, and unfavorable cosmetic outcomes being some examples of these downsides. In terms of cosmetic outcome, the mean of VAS score for total participants showed satisfactory results. As expected, patients with slide-swing flap showed significant difference in cosmetic satisfaction compared with the secondary method. Cosmetic appearance of the scar after procedure should be carefully taken into consideration, since PSD mostly involves young-aged patients. Actually, previous studies stated the fact that PSD is more prevalent amongst young-aged men [15]. Our results also demonstrated the same findings. We observed that PSD is more prevalent amongst men compared with women, with a male to female ratio of nearly 6:1 and a mean age of 29.15 ± 8.36 years old. It is noteworthy to mention that the main reason behind this gender discrepancy thought to be the role of male sex hormones in PSD pathogenesis [16].

Apart from the cosmetic outcomes, post-operation pain severity is of great importance. Our study showed that the pain severity in patients underwent slide-swing plasty was significantly less compared with the other group, especially in the first week of post-operation. Interestingly, previous studies comparing the pain severity of secondary closure with Limberg procedure demonstrated that secondary healing group was less than the Limberg flap [17]. Such differential tendencies are mainly attributed to the extensive dissection of Limberg flap. However, we concluded that despite longer operation time of slide-swing flap (compared with secondary closure), the pain severity reduced significantly in the trial group. Also, since the excision is mainly limited to the defect border the recovery time would be also relatively quicker. As we observed in our study, recovery time was significantly faster compared with the secondary closure of PSD. Similar to the literature [18], our data supported that slide-swing procedure compared with secondary closure, shortens the duration of incapacity for work and improved patients’ life style significantly. Maghsudi et al. concluded that secondary closure of PSD has the worst outcome in terms of healing time, and incapacity for work compared with primary closure with/without Limberg flap [14].

Recurrence of PSD following surgical management depends on various factors including BMI, prolonged sitting, and chronic irritation. However, the most important factor attributed to decrease incidence of PSD recurrence is complete excision of the sinus tracts [19]. Results of our study demonstrated that both surgical techniques showed satisfactory outcome in terms of recurrence, with only one (2.0%) case in the slide-swing group and two (4.0%) patients in the secondary group had recurrence of primary disease. These findings were similar to previous studies. For instance, an RCT by Arnous et al. [11] was conducted on 31 patients undergoing Limberg flap, and showed no recurrence at the end of follow-up sessions. Although, the data regarding recurrence rate of PSD varies widely depending on the surgical procedure. The recurrence rate of 0 to 3% has been documented for the flap procedures especially the Limberg flap technique [20-21]. That said, primary closure without flap technique is associated with the highest rate of recurrence with a range of 7%-42% [22-24]. Secondary closure on the other hand, due to the extensive dissection of this procedure, provides much better outcomes in terms of disease recurrence [23]. 

A thorough look at these two surgical techniques complications, we found that despite administration of pre/post-operation antibiotic, wound infection still occurred in both groups which was in accordance with the literature. Previous studies reported the rate of wound infection of 6%-20% after PSD surgeries [14]. Although some studies reported a significant difference regarding wound infection between different surgical techniques [25], the majority of published articles found insignificant difference on the matter [26-28]. Also as expected, we observed that wound hematoma tends to occur more frequent in the swing flap compared with the controls. We hypothesized that longer duration of operation in the swing flap technique along with the open wound nature of secondary healing technique, were the main reasons behind this finding. In our study seroma formation, a serous fluid accumulation under the skin after surgical interventions was observed with a much higher incidence rate compared with the literature. Almost all cases of secondary closure developed with seroma formation after the first week of surgery, which was significantly more compared with slide-swing flap patients. Arslan et al. reported the incidence rate of 5.2%, 7.4%, and 19.8% for seroma formation in Limberg flap, modified Limberg flap and Karydakis flap, respectively [27]. Few studies suggested that application of suction drains may lead to decreased seroma formation [25, 29]. However, there are various studies disagreeing with this statement, as drains have minimal role in preventing seroma formation and in fact could increase the need for analgesics and hospitalization [30].

This survey is limited by the relatively low sample size of participant included. Despite this limitation, this study has one of the largest series evaluating patients with PSD undergoing surgical intervention. Another limitation to this study is that despite blinded randomization of participants, patients in slide-swing flap technique had shorter duration of disease prior to surgery, compared with the secondary closure. Thus, a confounder bias should be taken into consideration. The third limitation of this study is the single-blinded nature of this RCT because the surgeon was aware of the study protocol. Despite these limitations, this study has many strengths. To the best of our knowledge, this article is the first survey describing the slide-swing flap technique as an option for surgical management of PSD. In addition to its novelty, throughout our survey the entire surgical interventions were carried out by one experienced surgeon in order to eliminate the risk of instrument bias. Also, the authors determined to evaluate all possible factors that could lead to improve patient’s outcome and recurrence of disease. 

## Conclusions

In this RCT, the authors introduced slide-swing flap as an alternative surgical technique for treatment of PSD and compared this method with the secondary closure. Compared with secondary closure, the slide-swing flap was associated with excellent cosmetic outcome, recurrence of the disease, and recovery time. Also, the post-operative complications were significantly lower compared with the traditional method. Compared with other off-midline flap techniques the swing flap seems to be the superior surgical option, mainly due to its technical simplicity, adjustable incision line, and lack of unnecessary skin excision.
